# Silymarin protects plasma membrane and acrosome integrity in sperm treated with sodium arsenite

**Published:** 2016-01

**Authors:** Farzaneh Eskandari, Hamid Reza Momeni

**Affiliations:** *Department of Biology, Faculty of Science, Arak University, Arak, Iran.*

**Keywords:** *Spermatozoa*, *Arsenic*, *Silymarin*

## Abstract

**Background:**

Exposure to arsenic is associated with impairment of male reproductive function by inducing oxidative stress. Silymarin with an antioxidant property scavenges free radicals.

**Objective:**

The aim of this study was to investigate if silymarin can prevent the adverse effects of sodium arsenite on ram sperm plasma membrane and acrosome integrity.

**Materials and Methods:**

Ram epidydimal spermatozoa were divided into five groups: spermatozoa at 0 hr, spermatozoa at 180 min (control), spermatozoa treated with silymarin (20 μM) + sodium arsenite (10 μM) for 180 min, spermatozoa treated with sodium arsenite (10 μM) for 180 min and spermatozoa treated with silymarin (20 μM) for 180 min. Double staining of Hoechst and propidium iodide was performed to evaluate sperm plasma membrane integrity, whereas comassie brilliant blue staining was used to assess acrosome integrity.

**Results:**

Plasma membrane (p< 0.001) and acrosome integrity (p< 0.05) of the spermatozoa were significantly reduced in sodium arsenite group compared to the control. In silymarin + sodium arsenite group, silymarin was able to significantly (p< 0.001) ameliorate the adverse effects of sodium arsenite on these sperm parameters compared to sodium arsenite group. The incubation of sperm for 180 min (control group) showed a significant (p< 0.001) decrease in acrosome integrity compared to the spermatozoa at 0 hour. The application of silymarin alone for 180 min could also significantly (p< 0.05) increase sperm acrosome integrity compared to the control.

**Conclusion:**

Silymarin as a potent antioxidant could compensate the adverse effects of sodium arsenite on the ram sperm plasma membrane and acrosome integrity.

## Introduction

Human activities have altered the global cycle of heavy metals and metalloids, including the toxic non-essential elements like arsenic (1). Although arsenic is identified as a major global health hazard, its derivatives are still used extensively in the industry and the agriculture (2). Arsenic is also used in drugs, herbicides, insecticides not only contaminate environment but also endangers human health by entering into food chains (3-6). 

Serious developmental effects, cancer, and cardiovascular diseases have been associated with long-term exposure to arsenic in human (7). Arsenic can impair male reproductive function. The reduction of spermatogenesis and released testosterone (8) as well as inhibiting the functions of testicular enzyme activity were reported as effects of arsenic toxicity in rodents (9). Arsenic also negatively induce a reduction in the weight of male sex organs, sperm number, viability and motility and can also increase the number of abnormal spermatozoa (10-13). Several line of studies suggest that arsenic exerts its toxicity by generating reactive oxygen species (ROS) (14).

Oxidative stress induced by free radicals could in turn disturb the membrane structural components which lead to the plasma membrane dysfunction. The products of medical plants are known to exert their protective effects by scavenging free radicals and modulating the antioxidant defense system. Therefore, the application of natural antioxidants could be an effective approach for the reduction of oxidative stress in the body. Silymarin, a natural polyphenolic flavonoid, is extracted from the seeds of silybum marianum also called “milk thistle” (15). This flavonoid is known as a potent antioxidant which exerts its protective role in wide variety of cells against the oxidative stress (16, 17).

Male infertility is not only related to a normal semen analysis. Instead, it could be due to a disturbance in functional competence of the sperm membranes. The integrity of sperm plasma membrane and its function is a crucial factor for the sperm metabolism, capacitation, ova binding and acrosome reaction. Acrosome integrity is also required for the penetration of the sperm into the zona pellucid and fertilization. Thus, the assessment of plasma membrane and acrosome integrity could be useful for predicting the fertilizing ability of the sperm. 

The present study was done to explore whether silymarin, as a potent antioxidant, can prevent the adverse effect of sodium arsenite on plasma membrane and acrosome integrity of ram sperm.

## Materials and methods

**Epididimal sperm collection and treatments**

In this experimental study, adult Farahani's ram testes (n=6 from different animals) were received from arak slaughterhouse and transferred to the research laboratory on ice (4^o^C) immediately after ram daily slaughter for public consumption. The ethical issues in the use of animals in research were observed. A few longitudinal incisions were performed on the cauda epididymis and spermatozoa were then washed into a sterile falcon by a syringe containing Ham's F10 medium (Sigma, USA). Sperm number and sperm motility were determined according to the World Health Organization guidelines in order to evaluate the sperm quality (18). 

High quality sperm samples were then used for further experiments. The sperm samples were separated in eppendorf tubes as each one contained 5×10^6^ spermatozoa and was divided into five groups (n=6 per group): 1. Spermatozoa at 0 hr, 2. Control spermatozoa, 3. Spermatozoa treated with sodium arsenite (10 μM, Merck, Germany), 4. Spermatozoa treated with silymarin (20 μM, Sigma, USA) + sodium arsenite (10 μM) and 5. Spermatozoa treated with silymarin (20 μM). The samples 2-5 were kept at 37^o^C in a CO_2_ incubator for 180 min.

**Evaluation of sperm plasma membrane integrity**

Sperm plasma membrane integrity was assessed with Hoechst 33342-Propidium iodide (PI) double staining (19). Briefly, 5μl of Hoechst 33342 (5 μg/ml; Sigma, USA) was added to each tube containing sperm suspension in the medium and incubated at 37^o^C for 15 min. Next 50 μl of PI (50 μg/ml; Sigma, USA) was added to the suspension and incubated at 37^o^C for 5 min. A 20 μl of the sperm suspension was placed on a glass slide, mounted in the glycerol/PBS solution (1:1) and cover slipped. The slides were then examined under an Olympus fluorescence microscope using the appropriate excitation and emission filters. 

In this method, two populations of the sperm could be distinguished; the first group which consisted of damaged sperm plasma membrane with the PI red staining over the head and the second population which contained intact sperm plasma membrane indicated Hoechst blue staining over the head. One hundred spermatozoa were counted in at least 5 different microscopic fields per slide and expressed as the percentage of sperm plasma membrane integrity.

**Evaluation of sperm acrosome integrity**

Sperm acrosome integrity was evaluated using coomassie brilliant blue staining (20). In brief, sperm samples were transferred onto a glass slides; thin smear was prepared and air-dried. The smears were fixed with 5% paraformaldehyde in phosphate-buffered saline (PBS, pH=7.4) for 15 minutes and washed with PBS. The samples were stained with 0.25% coomassie brilliant blue (MERCK, Germany) in 10% glacial acetic acid and 25% methanol for five minutes. 

The slides were washed with distilled water. The stained slides were then observed under a light microscope at 1000× magnification. This method stains the acrosomal cap blue in acrosome-intact spermatozoa but does not stain the acrosome region in damaged acrosome spermatozoa. At least 100 spermatozoa were counted in five different microscopic fields per slide and expressed as the percentage.

**Statistical analysis**

The results are expressed as mean±SD for six tests from different rams per group. One-way analysis of variance (ANOVA) followed by Tukey's test was used to assess the statistical significance of the data and p< 0.05 was considered significant.

## Results

The integrity of sperm plasma membrane

**Figure 1:**
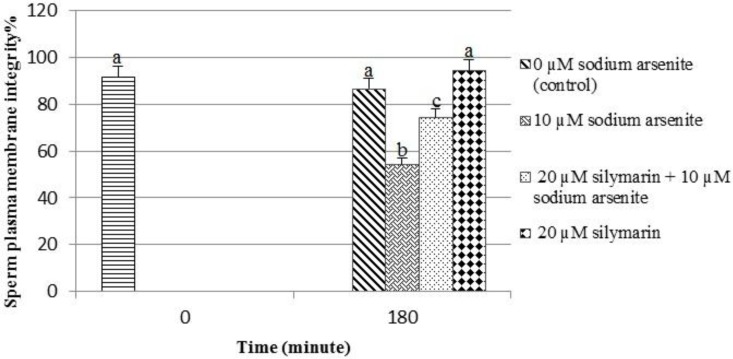
Evaluation of ram sperm plasma membrane integrity using Hoechst-Propidium iodide double staining. Means with the same words do not differ significantly. Mean±SD, one way ANOVA, Turkey’s test, n=6 per group, (p< 0.05).

**Figure 2:**
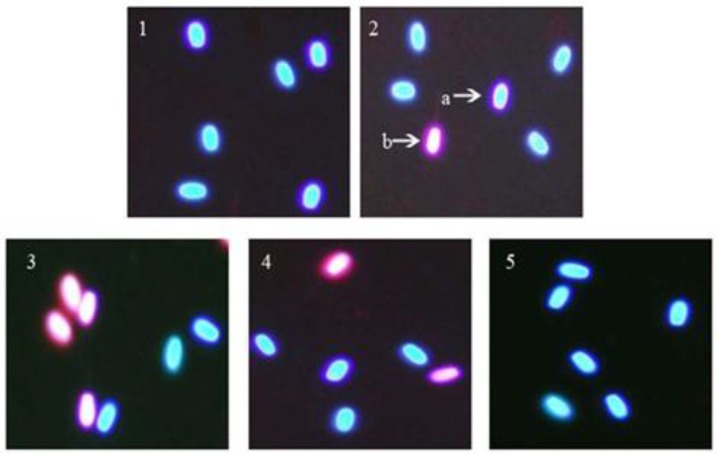
Evaluation of ram sperm plasma membrane integrity using Hoechst-Propidium iodide double staining. 1. Spermatozoa at 0 hour, 2. Spermatozoa at 180 min (control), 3. Spermatozoa treated with sodium arsenite (10 μM) for 180 min, 4. Spermatozoa treated with silymarin (20 μM) + sodium arsenite (10 μM) for 180 min and 5. Spermatozoa treated with silymarin (20 μM) for 180 min. Magnification 1000×. a) Sperm head with intact plasma membrane stained blue. b) Spermatozoa with red head are representative of damaged sperm plasma membrane

The percentage of sperm plasma membrane integrity in the sodium arsenite group (54.25%) was significantly (p<0.001) decreased compared to the control group (spermatozoa in 180 min) (86.7%). In the silymarin + sodium arsenite group (74.5%), silymarin could significantly (p< 0.001) reverse the adverse effect of sodium arsenite on sperm plasma membrane integrity compared to the sodium arsenite group (54.25%) (Figure 1, 2).

**The integrity of sperm acrosome**

**Figure3:**
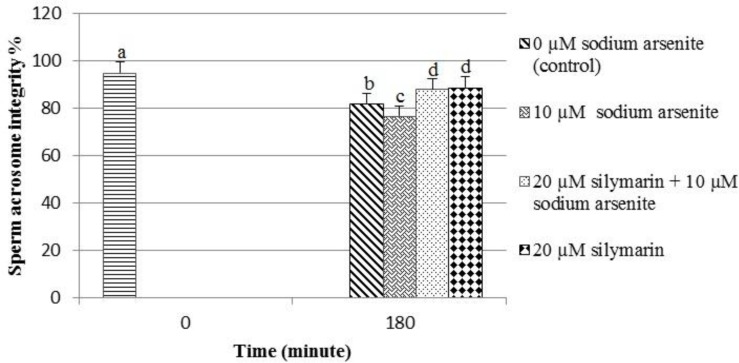
The integrity of ram sperm acrosome evaluated by coomassie brilliant blue staining. Means with the same words do not differ significantly. Mean±SD, one way ANOVA, Turkey’s test, n=6 per group, (p<0.05).

**Figure 4:**
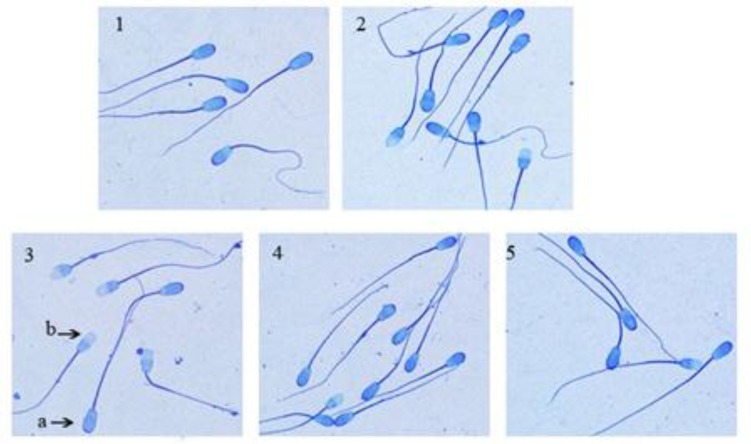
Evaluation of ram sperm acrosome integrity using coomassie brilliant blue staining. 1. Spermatozoa at 0 hour, 2. Spermatozoa at 180 min (control), 3. Spermatozoa treated with sodium arsenite (10 μM) for 180 min, 4. Spermatozoa treated with silymarin (20 μM) + sodium arsenite (10 μM) for 180 min and 5. Spermatozoa treated with silymarin (20 μM) for 180 min. Magnification 1000×. a) Sperm with intact acrosome displayed blue acrosomal cap. b) Damaged sperm acrosome with colorless acrosome region

The application of sodium arsenite significantly (p< 0.05) decreased the percentage of the sperm acrosome integrity (76.82%) compared to the control (82.15%). In the silymarin + sodium arsenite group (88%), silymarin could significantly (p< 0.001) reverse the adverse effect of sodium arsenite on acrosome integrity compared to the sodium arsenite group (76.82%) (Figure 3, 4). The incubation of spermatozoa for 180 minutes in the control group caused a significant (p<0.001) decrease in the percentage of sperm acrosome integrity (82.15%) compared to spermatozoa at 0 hour (95%). In addition, in spermatozoa treated with silymarin alone (20 μM for 180 minutes), the percentage of acrosome integrity (88.75%) was significantly (p< 0.05) increased compared to the control (82.15%).

## Discussion

In the present study we tested the adverse effects of sodium arsenite on plasma membrane and acrosome integrity of ram epididymal sperm. We also examined the protective effect of silymarin on these parameters in sodium arsenite treated spermatozoa. The fertilizing potential of sperm depends on its functional competence. Sperm plasma membrane and acrosome integrity play an important role for fertilizing capacity. A dysfunction in the sperm membrane integrity could therefore seriously affect male infertility.

Our results showed a significant decrease in the percentage of plasma membrane and acrosome integrity in sodium arsenite treated spermatozoa. Oxidative stress induced by arsenic through the formation of ROS and glutathione (GSH) depletion is likely to damage sperm membranes. In mature spermatozoa, the high concentration of unsaturated lipids is associated with a low level of antioxidant enzymes which make them highly susceptible to lipid peroxidation (LPO) induced by oxidative attack (21-23). We therefore hypothesized that toxic effects of sodium arsenite on the sperm plasma membrane and acrosome integrity could be due to the ability of this toxicant in the induction of oxidative stress followed by lipid peroxidation. If our hypothesis was true, the application of an antioxidant should reverse hazardous effect of sodium arsenite on these sperm parameters. We showed that in spermatozoa treated with silymarin + sodium arsenite, silymarin as a potent antioxidant, could significantly compensate the adverse effects of sodium arsenite on the percentage of membrane and acrosome integrity of these spermatozoa compared to the sodium arsenite group (24). Therefore, it could be speculated that silymarin by improving the activity of the sperm antioxidant defense system exerted its antioxidant role on the sodium arsenite mediated toxicity. The assessment of indicators of lipid peroxidation such as malondialdehyde level in the mentioned groups is suggested to provide insights toward this possible mechanism. In mammalian spermatozoa, acrosome reaction depends on the increase in intracellular Ca^2+^ concentration (25). Since arsenic is able to induce Ca^2+^ rise in the cells, it is also possible that sodium arsenite by increasing intracellular Ca^2+^ concentration is involved in spontaneous sperm acrosome reaction (26). 

The incubation of spermatozoa for 180 min (control group) caused a significant decrease in the percentage of acrosome integrity compared to spermatozoa at 0 hr. The Na^+^/K^+^-ATP-ase plays an important role in maintaining the acrosomal structure in ram spermatozoa (27). Therefore, one possibility for the decreased acrosome integrity over the incubation time might be associated with ATP reduction. Another possibility could be due to the lipid peroxidation induced by free radicals or an imbalance between oxidating and antioxidant defense system. Since the application of silymarin alone for 180 min significantly increased the percentage of acrosome integrity compared to the control, it is therefore possible that the induction of acrosome integrity during the incubation time attributed to oxidative stress. This effective role of silymarin might be associated with its antioxidant effects in improving the capacity of sperm antioxidant defense system.

## Conclusion

Our results indicate that sodium arsenite has a negative influence on sperm plasma membrane and acrosome integrity. On the other hand, silymarin is able to compensate the adverse effects of sodium arsenite on these sperm parameters.
